# Data from RE distressed market: Properties auctions in Italy

**DOI:** 10.1016/j.dib.2018.03.009

**Published:** 2018-03-08

**Authors:** Rubina Canesi, Giuliano Marella

**Affiliations:** a Department of Earth and Environment, Boston University, Boston, MA, USA; b ICEA - Civil, Environmental and Architectural Engineering - University of Padova, Padova, Italy

**Keywords:** Distressed market, Real estate auction, Performance, Survey, Italy

## Abstract

This paper reports data describing Real Estate (RE) distressed market, focusing on properties foreclosures occurred in North-East Italy. A survey was carried out consulting financial institutions, courts of law and different associations of public notaries. The aim of this survey was to record RE auctions, collecting technical and socio-economic features. The novelties of this survey are mainly two. The first consists in the dataset itself, due to the difficult in collecting such type of data in the Italian scenario. The second one is the recording of socio-economic features related to the occurrence of the survived Re auction. The collected socio-economic characteristics regard housing market trends and performance as well as demographic features. These features could be analyzed in order to relate the performances of this type of distressed market and the surrounding urban context. The database come from an analysis of the authors regarding the discount existing between the Forced Sale Price and the Market value, assessed by appraisers.

**Specifications table**

**Table:** 

Subject area	Economics
More specific subject area	Real Estate
Type of data	Table
How data was acquired	Survey
Data format	Raw
Experimental factors	Sample pretreatment: observations with many incomplete data have been rejected. The survey was limited only in one Italian region, in order to avoid upward biased due to spatial price interdependence. The surveyed variables have been measured both in nominal and in ordinal scales.
Experimental features	We conduct the survey collecting data from financial institutions, courts of law and different associations of public notaries, all placed in North-East Italy. Records reflect socio-economic conditions of the surrounding areas, physical and profitability characteristics of the auctioned properties, and features related to the performing of the auction market.
Data source location	Region: Veneto; Italy.
Data accessibility	Data are with this article

**Value of the data**

• The presented data is the first to be published on the Italian auction market. There are no other public databases that describe this distressed market.
• The records describe auctioned properties surveying both technical and socio-economic features, affecting and/or effected by the auction performance.
• The collected data are easy to interpret and can be processed by qualitative and quantitative statistical analysis, e.g., rough set analysis and hedonic regression models.
• Demographic data and RE auction performance are demonstrated to be related by international [1], but no evidence is still carried out in the Italian context.

## 1 Data

This database denotes the main novelty of the paper. In the Italian milieu, all data on RE auction market and foreclosure procedures are collected by associations of public notaries and courts of law in hardcopy archive. Therefore, this survey sheds light on the Italian RE auction market, on its procedure and on the context features, in which it takes place [2].

We collected data on RE auction procedures by cooperating with associations of public notaries in the Veneto Region. Our database is composed by 125 forced sale properties in the North-East Italy, auctioned between 2008 and 2016. Table 1 lists the selected surveyed characteristics, selected both by consulting literature [3], [4], [5], [6], [7] and according to the aims of our survey. All the features are clustered into four different groups (Physical Features-PhF, Profitability Features-PF, Socio-Economic Features-SEF, Auction Market Features-AMF), classified by an Identification Code (ID), and identified by a Coding System.

**Table 1:** Surveyed variables.

**Cluster**	**ID**	**Characteristic**	**Measure scale**	**Coding system**
*Physical Features (PhF).*	TC	Type Classification	Ordinal	0: factory, 1: residence, 2: retail, 3: mixed, 4: build-on land, 5: office, 6: agricultural building
	GBA	Gross Building Area	Continuous	m^2^
	Q	Quality	Ordinal	1: poor, 2: adequate, 3: fairly good, 4: good, 5: excellent
	SM	State of Maintenance	Ordinal	1: poor, 2: adequate, 3: fairly good, 4: good, 5: excellent
			
*Profitability Features (PF)*	Oc	Occupancy	Ordinal	0: unoccupied, 1: tenant, 2: owner
				
*Socio-Economic Features (SEF)*	Loc	Physical Location	Ordinal	0: center, 1: semi-center, 2: suburban
	Inc	Income	Continuous	€
	ΔNTN	Variation of NTN	Percent	%
	REAI	RE Activity Index	Percent	%
	Pop	Population	Rational	Number
				
*Auction Market Features (AMF)*	DD	Days on the market	Rational	gg
	NA	Numbers of auctions	Rational	Number
	Ds	Discount	Percent	%
	Pr	Premium	Percent	%
	DV	Date of value	Date	month/year

## 2 Experimental design, materials and methods

We restricted the survey both limiting the temporal extension and the geographical dispersion, thus ensuring some degree of homogeneity [8], [9], [10], [11], [12]. Therefore, we focused the sampling in a restricted area, in order to avoid several variables relating to purely territorial dynamics [13], [14], [15]. We also surveyed data for an eight years period, after the big financial collapse in 2016.

For each auctioned property we surveyed the information listed in Table 2 by consulting the archives of the above-mentioned institutions. We collected data on physical characteristics of the properties (such as typology classification, legal description, size, pictures, quality of constructions and state of maintenance), but also data connected to the distressed market where they were sold (such as fair market value, date of sale, date of value, estimated value, methodological approach and number of bidding proceedings).

**Table 2:** Survey chart.

**Surveyed characteristics**	**Unit of measure/classification/description**
N° of auction	N°
Synthetic description	……
Legal description	Fg. …, Mapp. …, Sub. …, Cat. …, Cl. …, R. …
Address	……
Type classification	factory, residence, retail, mixed, build-on land, office, agricultural building
Gross building area	m^2^
Occupancy	empty (unoccupied), occupied by tenant, occupied by the owner
Pictures	Jpg.
Quality of constructions	poor, adequate, fairly good, good, excellent
State of maintenance	poor, adequate, fairly good, good, excellent
Date of value	././….
Estimated value	Euro
Methodological approach	Income Capitalization Approach / Sales Comparison Approach / Cost Approach
Fair market value	Euro
Date of sale	././….
Number of bidding proceedings	N°

We filled out all the survey charts, consulting local paper archives. Successively, we listed our database first summarizing the surveyed features and subsequently analyzing and processing some listed characteristics in new interpretative variables, clustered in SEF and AMF groups. The PhF, PF and AMF clusters consist of characteristics, which are chosen consulting international literature [2], [4], [16]. Instead, the features in SEF cluster are selected by the authors in order to capture and interpret socio-economic condition of the surrounding area, where the auction takes place [17], [18], [19].

Furthermore, we cluster the data in the following groups:

1) Physical Features (PhF): In this cluster we collected features able to describe the most relevant characteristics of the auctioned properties:
  – Type Classification (TC): An ordinal variable which describes the type of auctioned property, named 0 for factory type, 1 for residential property, 2 for retail, 3 for mixed space, 4 for development, 5 for office, and 6 for agricultural building;
  – Gross Building Area (GBA): A continuous variable recorded in square meter (m^2^).
  – Quality (Q): An ordinal variable used to describe the quality of the properties, including design work and materials employed. The coding system registers 1 for properties poor quality; 2 for adequate materials and finishing; 3 if they are fairly good; 4 if good, and 5 if the finishing is excellent.
  – State of Maintenance (SM): An ordinal variable used to describe the state of maintenance of the property, if it is new, recently renovated or damaged over the years. As Q, this variable is represented by qualitative judgments classifying in six different levels: 1 for poor SM usually in very old properties, never been renovated, 2 if the SM level is adequate, 3 if fairly good, 4 if good, and 5 if it is excellent and totally new.
2) Profitability Features (PF) is represented by an ordinal variable which describes the state of Occupancy of the auctioned property (Oc). This variable was selected in order to considered whether the house generates income. Indeed, Clauretie and Daneshvary [20] showed that the occupancy status influences foreclosure discounts. We survived if it is vacant (0), rented to tenants (1), or occupied permanently or occasionally by the former owner (2).
3) Socio-Economic Features (SEF): These variables represent, on the one hand, the property's physical location in relation to the city sprawl and, on the other hand, the socio-economic features that characterize the local market. These variables were selected to be able to interpret the activity of the involved market, in fact a Chow et al.’s study [21] explain that strong competitive market demand (when the number of bidders and transactions is large) leads also to higher forced sale prices. These variables could be useful in examining possible relationships existing between the performance of the competitive RE market and the high discounts (collected in AMF cluster) that effects the RE auction market.
  The surveyed features for this cluster are the following:
  – Physical Location (Loc): this variable considers the property's distance from the city center, by classifying it as central (0), semi-central (1) or suburban (2).
  – Income (Inc): this variable corresponds to the average per capita income, in €/year, for the city where the auction takes place. This data is published by the Italian Institute of Statistics (ISTAT).
  – Variation of the number of local real estate transactions (ΔNTN): this percentage index describes the activity of the local real estate market. It is the percentage variation in the number of local real estate transactions (NTN) over the observation period.
  – RE Activity Index (REAI): this percentage index describes the activity and the performance of the local real estate market. This index is represented by the ratio between NTN and the total stock of real estate units in the area where the property is auctioned.
  – Population (Pop): This rational variable measures the number of inhabitants in the city where the property is located.
4) Auction Market Features (AMF): In this cluster we calculated the following variables from the surveyed data (Table 2), in order to describe the performance of the Italian RE auction market:
  – Days on the market (DD): the number of days a property stays on the auctioned market before being sold. This variable is calculated as the difference between the date of sale (closing date) and the first auction starting date (opening bid).
  – Numbers of auctions (NA): the number of bidding proceedings before the selling.
  – Discount (Ds): this variable represents the percentage variation between the first listing value (which equal to the market value assessed by the appraisal) and the selling price, which the property is sold. It is important to stress that, to compare the values over time, all the selling price were discounted before the calculation of the Ds, in order to avoid temporal bias. We estimated a Real Estate price index, consulting the RE quotations archives published by Consulente Immobiliare [22], to discount the selling prices. We developed a matrix for each surveyed province that covers the entire period from 2008 until 2016.
  – Premium (Pr): this variable represents the premium paid by the winner bidder to win the auction. It is calculated as the percentage variation between the last listing value and the final selling price, which the property is sold. As for the Ds we discounted the selling price consulting the indexes matrix described above.
  – Date of value (DV): the date which refers the market value assessed by the appraisal.
